# The Effect of Periodontal Treatment on Hemoglobin A1c Levels of Diabetic Patients: A Systematic Review and Meta-Analysis

**DOI:** 10.1371/journal.pone.0108412

**Published:** 2014-09-25

**Authors:** Xingxing Wang, Xu Han, Xiaojing Guo, Xiaolong Luo, Dalin Wang

**Affiliations:** 1 Department of Stomatology, Changhai Hospital, The Second Military Medical University, Shanghai, China; 2 Department of Health Statistics, The Second Military Medical University, Shanghai, China; National Taiwan University, Taiwan

## Abstract

**Background:**

There is growing evidence that periodontal treatment may affect glycemic control in diabetic patients. And several systematic reviews have been conducted to assess the effect of periodontal treatment on diabetes outcomes. Researches of this aspect are widely concerned, and several new controlled trials have been published. The aim of this study was to update the account for recent findings.

**Methods:**

A literature search (until the end of January 2014) was carried out using various databases with language restriction to English. A randomized controlled trial (RCT) was selected if it investigated periodontal therapy for diabetic subjects compared with a control group received no periodontal treatment for at least 3 months of the follow-up period. The primary outcome was hemoglobin A1c (HbA1c), and secondary outcomes were periodontal parameters included probing pocket depth (PPD) and clinical attachment level (CAL).

**Results:**

Ten trials of 1135 patients were included in the analysis. After the follow-up of 3 months, treatment substantially lowered HbA1c compared with no treatment after periodontal therapy (–0.36%, 95%CI, −0.52% to −0.19%, *P*<0.0001). Clinically substantial and statistically significant reduction of PPD and CAL were found between subjects with and without treatment after periodontal therapy (PPD −0.42 mm, 95%CI: −0.60 to −0.23, *P*<0.00001; CAL −0.34 mm, 95%CI: −0.52 to −0.16, *P* = 0.0002). And there is no significant change of the level of HbA1c at the 6-month comparing with no treatment (–0.30%, 95%CI, −0.69% to 0.09%, *P* = 0.13).

**Conclusions:**

Periodontal treatment leads to the modest reduction in HbA1c along with the improvement of periodontal status in diabetic patients for 3 months, and this result is consistent with previous systematic reviews. And the effect of periodontal treatment on HbA1c cannot be observed at 6-month after treatment.

## Introduction

Periodontitis is a multi-factorial infectious disease of the soft tissues and bone that support the teeth [1], and it is a major cause of tooth loss in adults [2]. Periodontitis may lead to the development of a high systemic disease burden [3] and possibly affect general health [4]. It is reported that periodontitis is associate with rheumatoid arthritis [5]. cardiovascular disease [6] and even the patients in the periodontitis cohort exhibit a higher risk of developing oral cancer than those in the gingivitis cohort [7].

Diabetes mellitus is a chronic, non-communicable disease and also one of the major global public health issues [8]. The distinguishing features of diabetes mellitus type 1 and type 2 are autoimmunity and chronic low-grade inflammation, respectively [9]. A large number of studies support the point that there is an association between diabetes and periodontal disease [10]: Firstly, individuals with diabetes have a higher prevalence of periodontitis [11]–[13]. Nelson et al. [14] reported that the incidence of periodontitis was 2.6 times higher in subjects with diabetes than those without. Tsai et al. [11] reported that patients with poorly controlled diabetes have a 2.9 times higher risk to have sever periodontitis compared to no-diabetic subjects. Secondly, diabetes mellitus can increase the severity of periodontitis [15]–[17] and the severity of the periodontitis is always greater than individuals without diabetes [10], [11]. And periodontitis is considered to be one of the diabetic complications [18], [19].

Although strong evidence tends to support the adverse effects of diabetes on periodontitis, numerous reports indicate that periodontitis can adversely affect glycemic control in diabetics [20], [21]. It is considered that this close association between periodontitis and diabetes is established on the reciprocal influence [22]. So a number of potential mechanisms for the two-way relationship between them have been proposed. It is known that periodontitis is a kind of inflammatory process which can generate localized and systemic infections, and emerging evidence implicates inflammation in the pathogenesis of type 2 diabetes [23], [24]. But the exact relationship between periodontitis and diabetes remains unclear.

Some intervention studies concerning the effects of periodontal disease on glycemic control of diabetic patients report more direct evidence. Iwamoto et al. [25] showed that antimicrobial periodontal therapy can cause a significant reduction of HbA1c value in type 2 diabetic patients. Gaikwad et al. [26] also found that scaling and root planning can improve glycemic control in patients with type 2 diabetes mellitus with or without adjunctive systemic doxycycline therapy. Bharti et al. [27] gave diabetic patients with periodontitis a periodontal treatment with topical antibiotics and found that this kind of therapy protocol can improve glycemic control and elevate serum adiponectin with improvement of periodontal status in type 2 diabetic patients. However, a recent phase III randomized controlled clinical trial (RCT) with 514 patients by Engebretson et al. [28] revealed that nonsurgical periodontal therapy did not improve glycemic control in patients with type 2 diabetes.

HbA1c is related to the mean blood glucose concentration over the past 1–3 months [8], and it is also associated with an increased risk for diabetes complications [29]. So the periodic monitoring of HbA1c in diabetic patients was proposed [30]. It is considered to be a standardized measurement used to estimate the effect of diabetes treatment on control of glucose metabolism [31]–[33], and is widely used in trials to monitor diabetes status [34].

Several recent systematic reviews [35], [36] have been conducted to assess the evidence that periodontal treatment influences the level of HbA1c. Due to several new research reports with bigger sample size, we therefore performed this meta-analysis of RCTs to evaluate the effect of periodontal treatment on glycemic control of diabetic patients.

## Research Design and Methods

### Search strategy

Three databases, PubMed, Embase and Cochrane Library were searched according to a method previously described by Teeuw et al. [35] and Engebretson et al. [36], using identical search criteria and terms: ((periodontal disease) OR (periodont*[Text Word]) OR (periodontitis)) AND ((diabetes[Text Word]) OR (diabet*[Text Word]) OR (diabetic*[Title]) OR (diabetic patient*[Text Word]) OR (diabetes patient[Text Word]) OR (non insulin dependent diabetes) OR (niddm[Text Word]) OR (insulin dependent diabetes[Text Word]) OR (iddm[Text Word]) OR (type 1 diabetes) OR (t1 dm) OR (type 2 diabetes) OR (t2 dm)) AND ((therapy) OR (treatment) OR (intervention)) AND ((controlled clinical trial) OR (randomized controlled trial) OR (RCT)) AND (english[Language]) until the end of the January of 2014. Additional searches were conducted in PubMed’s medical subject headings with: (periodontal diseases) AND (diabetes mellitus) AND (therapeutics OR therapy OR intervention studies). Moreover, we searched the World Health Organization (http://www.who.int/triasearch) and Clinical Trials.gov (http://wwwClinicalTrials.gov.) Web sites for information on registered RCTs.

### Study selection criteria

The studies to be included in the systematic review had to meet the following criteria: (1) randomized controlled trial; (2) participants over the age of 18 with both diabetes (both type 1 and type 2) and periodontitis; (3) intervention consisting of non-surgical treatment with or without adjunctive use of local drug delivery and systemic antibiotics; (4) comparator group with no periodontal treatment or delayed treatment; (5) study duration ≥3 months; (6) outcome consisting of mean change in HbA1c level, or pre- and post-treatment HbA1c levels.

Two independent reviewers (Xingxing Wang and Xiaolong Luo) evaluated all retrieved articles. Titles and abstracts were scanned to rule out studies that did not meet inclusion criteria. From the selected articles, the full texts were reviewed followed by a decision on their eligibility for inclusion.

### Data extraction and quality assessment

Data extraction and quality assessment were independently conducted by two authors (Xingxing Wang and Xiaojing Guo) using a standardized approach, and disagreements were adjudicated by a third reviewer (Dalin Wang) after referring back to the original articles.

Data retrieved from the studies included publication details (year of publication, name of first author and country) and trial characteristics (Patients’ types and treatment of diabetes, study design, sample size, interventions, follow-up duration, change of HbA1c and periodontal parameters). The quality of the included studies was assessed by Risk of Bias tool according to the Cochrane handbook for systematic reviews of interventions (Version 5.1.0) [37].

### Statistical analyses

The absolute difference of HbA1c percentage in each treatment arm was recorded. When not reported, it was calculated for the intervention and the control groups by means of the formula [35]:
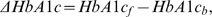
where HbA1c_f_ is the mean HbA1c value after treatment and HbA1c_b_ is the mean HbA1c value before treatment. And if the standard deviation was not reported, it was obtained by the formula [35], [38]:
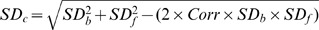
Where SD_c_ is the standard deviation of mean change of HbA1c, SD_b_ is the standard deviation of mean baseline HbA1c values, SD_f_ is the standard deviation of mean end HbA1c values. Corr is the correlation between the baseline and end values, it was set at 0.5, consistent with the previous reviews [35], [38].

For the three-arm studies, the two treatment groups or the two control groups, which were assumed to be “group 1” and “group 2”, were firstly combined into a single group according to the “Cochrane handbook for systematic reviews of interventions (Version 5.1.0)” [37] and “Introduction to meta-analysis” [39].

Sample size: 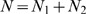


Mean: 
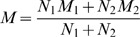


SD: 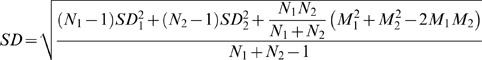


For periodontal clinical parameters, according to the Cochrane handbook for systematic reviews of interventions (Version 5.1.0) [37]: if the value of Corr less than 0.5 is obtained, then there is no value in using change from baseline and an analysis of final values will be more precise. We obtained the values of Corr by using the data extracted from Sun et al. 2011 [40] and Telgi et al. 2013 [8], and they were all less than 0.5, so we took the SD_f_ as the standard deviation of mean change of PPD and CAL.

The weighted mean difference (WMD) was calculated, Chi-square (χ^2^) and *I^2^* tests were used to assess the heterogeneity of the studies included in this meta-analysis. The heterogeneity of the trials will be regarded as low-level when *P*>0.10 for the χ^2^ test and *I^2^*<25%, and a fixed-effects model analysis was used to calculate a pooled effect; otherwise, a random-effects model was applied.

Forest plots showing the point estimate and confidence intervals for each study were created. Statistical significance was defined as a two-tailed *P*<0.05. All numerical data for meta-analysis were conducted using RevMan version 5.3 from the Cochrane collaboration.

## Results

### Study characteristics

**Figure 1:**
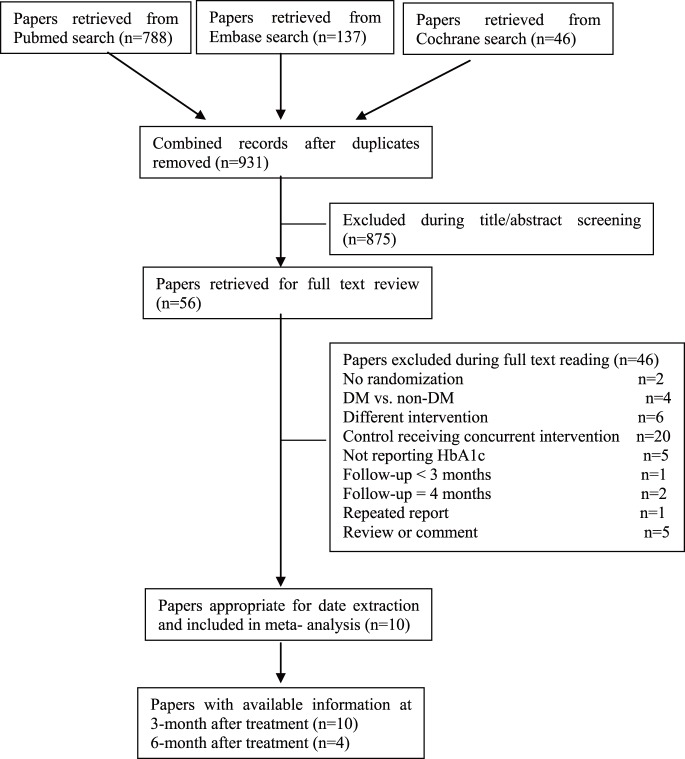
Flow diagram of the trials search and selection process.

The literature search resulted in 931 potentially relevant articles. The titles and abstracts of these articles were scanned for relevance. And the second level full text search was initiated on those remaining studies. Fifty-six potentially eligible trials were identified for full-text review, 46 of which were excluded for specific reasons listed in Figure 1. The remaining ten trials of 1135 patients (614 in the treated group and 521 in the control group) [8], [28], [39]–[46] that met the inclusion criteria were included in the meta-analysis, and the characteristics of each study were shown in Table 1. Of the ten trials, seven studies [39], [41]–[46] were also included in the previous analysis [35], [36]. Whereas two other studies [48], [49] that were included in the previous systematic reviews [35], [36] were excluded because their study duration were 4 months and the outcomes of the 3 months were not available.

**Table 1:** Characteristics of included trials.

Author(s)	Country Number of subjects (tx, ctr) Single versus multicenter	HbA1c inclusion criteria Treatment in test and control group	Inclusion criteria	Periodontitis evaluation	HbAlc(%) (mean±SD) at baseline and at 3 months (mean change)	HbAlc(%) (mean±SD) at 6 months (mean change)	PPD (mm)(mean±SD), CAL (mm)(mean±SD) at baseline and at 3 months(mean change)	Number of subjects with diabetic medication at baseline Change in diabetic medication
Kiran M et al. 2005	Turkey 44DM2(tx:22 ctr:22) single center	HbA1c 6%–8% tx:SRP+OH ctr: no treatment	not reported	PPD, CAL, GR, PI, GI, BOP	tx:7.31±0.74∼6.51±0.80(–0.86) ctr:7.00±0.72∼7.31±2.08(0.31)	NA	tx:PPD 2.29±0.49∼1.80±0.25(–0.49) CAL 3.19±1.13∼2.80±1.03(–0.39) ctr:PPD 2.24±0.70∼2.26±0.63(0.02) CAL 2.92±1.10∼2.87±1.03(–0.05)	Diet control:3 Oral medication:30 Insulin:4 Insulin +oral:7 no change in the medication or diet
Singh S et al. 2008	India 45 DM2(tx1∶15 tx2∶15 ctr:15) single center	HbAlc not reported tx1:SRP TX2:SRP+doxycycline ctr:no treatment	30% or more of the teeth examined having ≥4 mm PD	PPD, CAL, PI, GI	tx1∶7.9±0.7∼7.3±0.6(–0.6) tx2∶8.3±0.7∼7.5±0.6(–0.7) ctr:8.08±0.7∼8.1±0.74(0.06)	NA	tx1:PPD 2.67±0.35∼2.33±0.35(–0.34) CAL 3.44±0.45∼3.14±0.45(–0.30) tx2: PPD 2.52±0.47∼2.14±0.46(–0.38) CAL 3.22±0.63∼2.88±0.61(–0.34) ctr:PPD 2.44±0.26∼2.40±0.46(–0.04) CAL 2.78±0.33∼2.83±0.35(0.04)	Baseline medication not reported no change in the medication or diet
Katagiri S et al. 2009	Japan 49 DM2(tx:32 ctr:17) multicenter	HbAlc 6.5–10 tx1:SRP+topical minocycline ctr:OH	≥11 teeth ≥2 sites with PD≥4 mm	PPD, BOP	tx:7.2±0.9∼7.06* ctr:6.9±0.9∼6.81	reported in a graph	tx:PPD 3.0±0.9∼2.2±0.5 CAL NA ctr: PPD 2.8±0.9∼2.6±0.7 CAL NA	Diet control:3 Oral medication:27 Insulin:19 anti-diabetic drugs were not changed
Koromantzos PA et al. 2011	Greece 60DM2(tx:30 ctr:30) single center	HbA1c 7.0%–9.9% tx:SRP+OH ctr: supragingival+OH	≥16 teeth, PD≥6 mm in≥8 sites, CAL≥5 mm, in≥2 quadrants	PPD, CAL, BOP, GI	tx:7.87±0.74∼7.14±0.54* ctr:7.59±0.66∼7.41±0.48	tx:(–0.72±0.93)* ctr:(–0.13±0.46)	tx:PPD NA CAL NA ctr:PPD NA CAL NA	Oral medication 48 insulin 19 change in medication reported
Sun WL et al. 2011	China 157DM2(tx:82 ctr:75) single center	HbA1c 7.5%–9.5% tx:SRP+OH+flap when indicated+antibiotics ctr: OH	≥20 teeth, PD>5 mm, CAL≥4 mm in ≥30% teeth or PD>4 mm, CAL>3 mm in ≥60% teeth	PPD, CAL, BI, PI	tx:8.75±0.67∼8.25±0.72(–0.50±0.18) ctr:8.70±0.65∼8.56±0.69(–0.14±0.12)	NA	tx:PPD 4.53±0.83∼2.97±0.78 (–1.15±0.66) CAL 4.85±1.38∼4.12±0.95 (–0.73±0.51) ctr:PPD 4.49±0.85∼4.28±0.81 (–0.21±0.19) CAL 4.88±1.39∼4.73±1.29 (–0.15±0.13)	Diet or oral medication no medication changes
Chen L et al. 2012	China 126DM2(tx1∶42 tx2∶43 ctr:41) single center	HbA1c not reported tx1:SRP+subgingival debridement at 3-month tx2:SRP+supragingival prophylaxis at 3-month ctr:no treatment+no OH	≥16 teeth, CAL≥1 mm	PPD, CAL, PI, BOP	tx1∶7.31±1.23∼7.30±1.50 tx2∶7.29±1.55∼7.43±1.53 ctr:7.25±1.49∼7.59±1.54	tx1∶7.09±1.34 tx2∶6.87±1.12 ctr:7.38±1.57	tx1:PPD 2.66±0.68∼2.27±0.50 CAL 3.57±1.31∼3.28±1.25 tx2:PPD 2.57±0.66∼2.20±0.39 CAL 2.95±1.21∼2.55±1.15 ctr:PPD 2.47±0.57∼2.38±0.47 CAL 3.37±1.24∼3.29±1.23	Diet control:4 Oral medication:109 Insulin:13 no medication changes
Moeintaghavi A et al. 2012	Iran 40DM2(tx:22 ctr:18) single center	HbA1c >7% tx:SRP+OH ctr: OH	not reported	PPD, CAL, PI, GI	tx:8.15±1.18∼7.41±1.18 ctr:8.72±2.22∼8.97±1.82	NA	tx:PPD 2.31±0.65∼2.21±0.6 CAL 3.14±1.08∼2.8±1.09 ctr:PPD 2.06±0.24∼2.33±0.3 CAL 3.1±1.05∼3.47±1.44	Oral medication Medical treatment unchange
Botero JE et al. 2013†	Colombia 39DM1, 66DM2(tx1∶33 tx2∶37 ctr:35) single center	HbA1c not reported tx1:scaling+azithromycin tx2:scaling+placebo ctr:supragingival prophylaxis+azithromycin	CAL≥4 mm in ≥2 interproximal sites, or PD≥5 mm in ≥2 interproximal sites	PPD, CAL, PI, BOP	tx1∶7.92±1.58∼±1.36(–0.8)* tx2∶7.98±2.16∼±1.91(–0.6) ctr:7.87±1.81∼±2.48(0.2)	tx1:(–0.4) tx2:(0) ctr:(0.2)	tx1:PPD 2.7±0.6∼2.3±0.6 CAL 2.8±0.8∼2.5±0.8 tx2:PPD 2.6±0.7∼2.5±0.5 CAL 3.1±1.16∼3.0±1.1 ctr:PPD 2.4±0.6∼2.2±0.4 CAL 2.9±1.1∼2.8±0.9	Baseline medication not reported change not reported
Telgi RL et al. 2013	India 60DM2(tx:20 ctr1∶20 ctr2∶20) single center	HbA1c not reported tx:scaling+CHX+brush ctr1:CHX+brush ctr2:brush	PD 4–5 mm, ≥28 teeth	PPD, PI, GI	tx:7.68±0.63∼7.10±0.64(–0.58±0.27) ctr1∶7.56±0.59∼7.31±0.59(–0.25±0.14) ctr2∶7.74±0.59∼7.75±0.58(0.004±0.12)	NA	tx:PPD 5.05±0.70∼4.59±0.72(–0.46±0.26) CAL NA ctr1:PPD 5.11±0.57∼4.87±0.55(–0.25±0.11) CAL NA ctr2:PPD 5.05±0.69∼5.03±0.69(–0.02±0.05) CAL NA	Oral medication change in medication not reported
Engebretson SP et al. 2013	USA 275DM2(tx:240 ctr:235) multicenter	HbA1c 7%–9% tx:SRP+CHX+OH ctr:OH	PD≥5 mm in ≥2 quadrants, ≥16 teeth	PPD, CAL, PI, BOP	tx:7.84±0.65∼(0.13) ctr:7.77±0.60∼(0.08)	tx:(0.15) ctr:(0.09)	tx:PPD 3.3±0.6∼2.8 CAL 3.5±0.8∼3.2 ctr:PPD 3.3±0.7∼3.2 CAL 3.5±0.9∼3.4	No diatetes medications:11 Oral medication:244 Insulin:80 Combination:179 Changes between treatment groups were similar

PI, plaque index; GI, gingival index; PPD, probing pocket depth; CAL, clinical attachment loss; GR, gingival recession; BOP, bleeding on probing; NA, not available; CHX, chlorhexidine gluconate; OH, oral hygiene; SRP, scaling and root planning.

*reported in a graph.

† data obtained by calculation.

All studies included were reported as RCTs, and the durations of the follow-up period were at least 3 months. Of the ten studies, five were of 6-month duration [28], [41]–[43], [45], but the data is not available in Katagiri et al. [43], we wrote to the author and we were not answered. And there was also one study was of 9-month duration [41]. All studies described a study population having type 2 diabetes and suffering from periodontitis, and also one study included type 1 diabetes [41]. Eight studies were single centre [8], [40]–[42], [44]–[47], two were multi-centered [28], [43]. The largest in terms of sample size of the study is Engebretson et al. [28] with 514 participants. Four trials were three-arm studies, [8], [41], [42], [47] and three of them [41], [42], [47] reported two intervention groups, while the other one [8] reported two control groups. And they were combined into a single group firstly.

**Figure 2:**
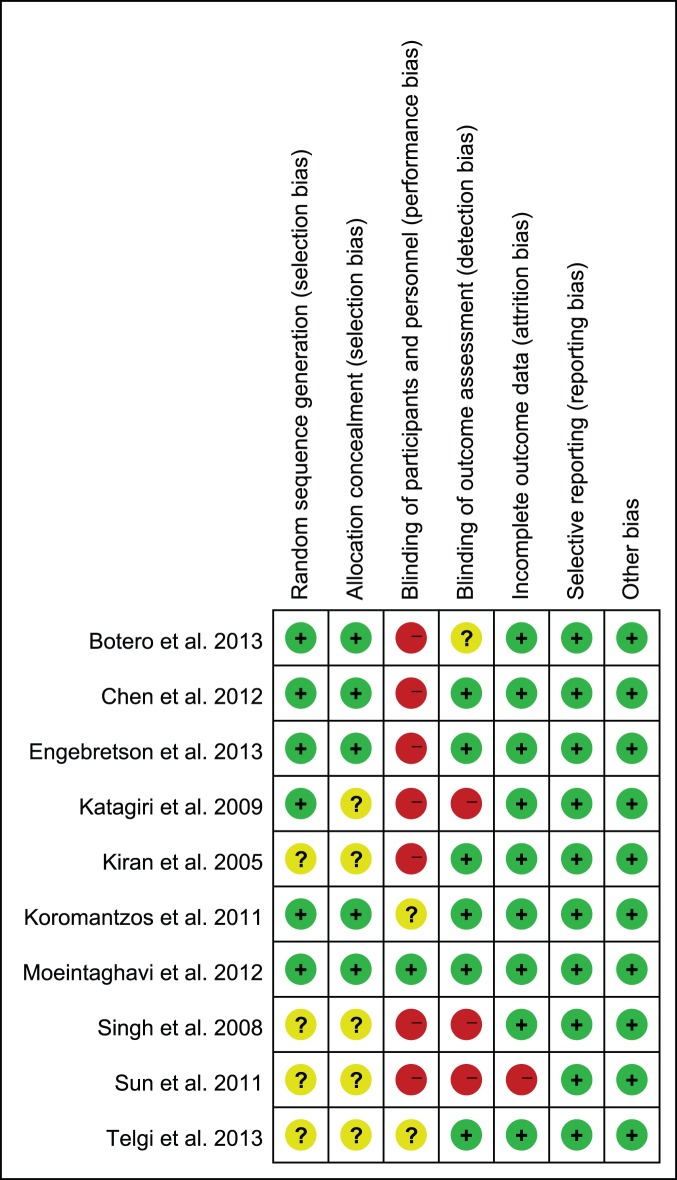
Judgements about each risk of bias item for each included study.

**Figure 3:**
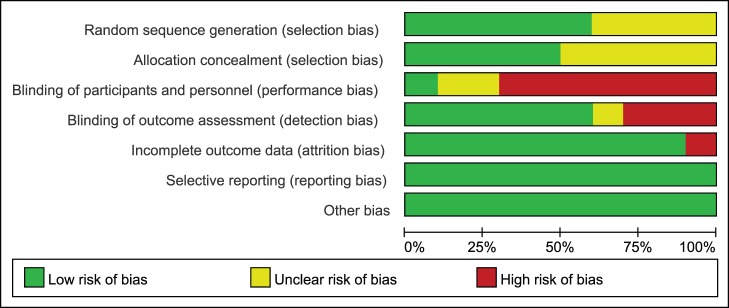
Each risk of bias item presented as percentages across all included studies.

Overall, all included studies had different levels of bias. Six studies [28], [41]–[43], [45], [46] showed a clear randomization scheme, the other four studies [8], [40], [44], [47] did not give out sufficient information about the generation of a randomized sequence. Concealment was inadequate in five of the included studies [8], [40], [43], [44], [47]. In one of the studies treatments were performed under the supervision of an expert [46]. Six studies were outcome assessment blinded [8], [28], [42], [44]–[46]. One study [46] excluded the data of the patients who did not finish the study at the baseline. (Fig. 2, 3).

**Figure 4:**
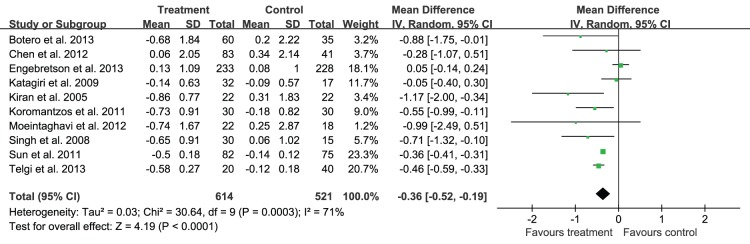
Forest plot presenting change in HbA1c (%) at 3-month.

Since the heterogeneity of every outcomes was assessed and shown as P for χ^2^ test and *I^2^*, as they were shown in Fig. 4, 5–7 that all of the P<0.10 for χ^2^ test and *I^2^*>25%, so there were heterogeneity among the included trials, and a random-effects model analysis was used. In addition, the results analyzed with the fixed-effects model, including the relevant forest plots (Fig. S1–S4), were provided as online supplements.

**Figure 5:**
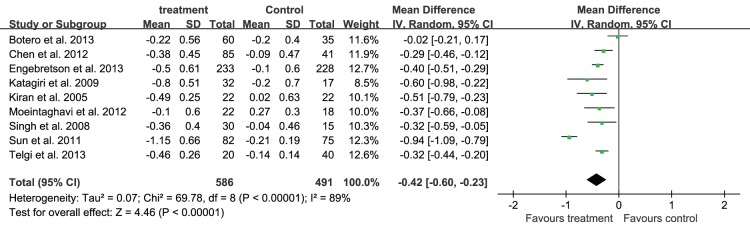
Forest plot presenting change in PPD (mm) at 3-month.

**Figure 6:**
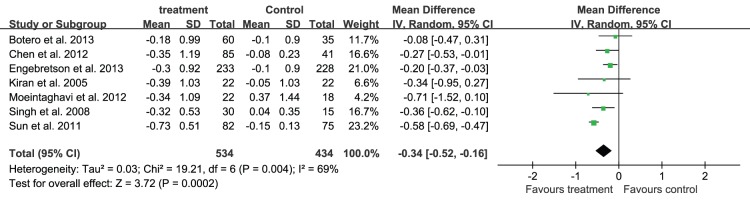
Forest plot presenting change in CAL (mm) at 3-month.

**Figure 7:**
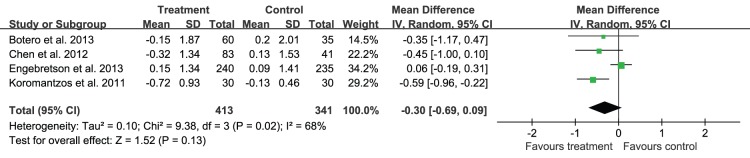
Forest plot presenting change in HbA1c (%) at 6-month.

### Change of HbA1c at 3-month

**Figure 8:**
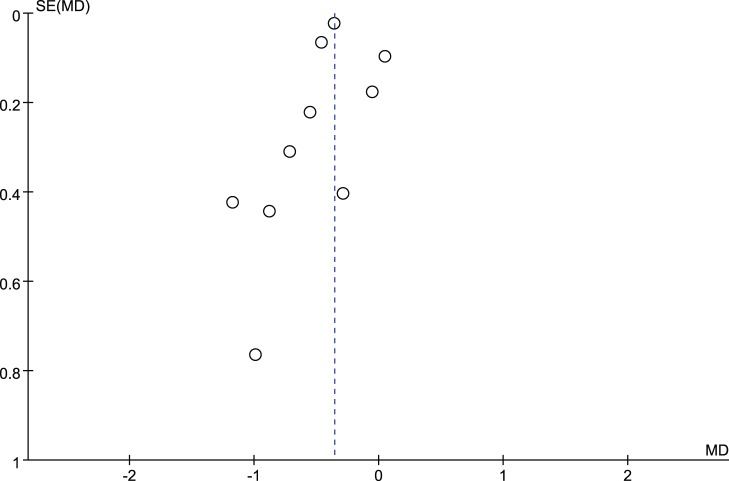
Funnel plot presenting change in HbA1c (%) at 3-month.

Data on change of HbA1c were available for analysis in 1135 patients enrolled in ten trials investigating the effect of non-surgical periodontal treatment on glycemic control of diabetic patients. The change of HbA1c for 3 months ranged from −0.86% to 0.13%, with a mean change of HbA1c from baseline of −0.36% (95%CI: −0.52%, −0.19%). As it was showed in Fig. 4, the forest plot described the effect on HbA1c in terms of mean reduction from baseline, and standard deviation, as a comparison between treatment and control groups. Nevertheless, there might be substantial heterogeneity in the change of HbA1c across studies (*P* = 0.0003, *I^2^* = 71%). Furthermore, publication bias may exist as the funnel plot (Fig. 8) displayed an asymmetrical distribution.

### Change of periodontal parameters at 3-month

Non-surgical periodontal treatment demonstrated a significant benefit on the periodontal status. Nine trials [8], [28], [40]–[44], [46], [47] reported the mean change of PPD in 1077 patients. The mean change of PPD from baseline to the 3-month after treatment was −0.42 mm (95%CI: −0.60, −0.23) (Fig. 5); seven trials [28], [40]–[42], [44], [46], [47] reported the mean change of CAL in 968 patients, and the mean change of CAL 3 months after treatment was −0.34 mm (95%CI: −0.52, −0.16) (Fig. 6).

### Change of HbA1c at 6-month

Data on the change of HbA1c for 6 months in diabetic patients with periodontitis were available for 754 patients enrolled in four trials [28], [41], [42], [45]. The change of HbA1c for 6 months ranged from −0.72% to 0.15%, and the result presented no statistical difference between the treatment and control groups (–0.30%, 95%CI: −0.69 to 0.09, *P* = 0.13), and there might be heterogeneity in the change of HbA1c for 6 months across studies (*P* = 0.02, *I^2^* = 68%) (Fig. 7).

### Treatment modalities

All treatment group interventions consisted of non-surgical periodontal therapy with or without adjunctive topical or systemic antibiotics, and/or topical antiseptics. Six trials [28], [40], [43]–[46] were two-arm studies, and four trials [8], [41], [42], [47] were three-armed. Of the three-arm studies, Boter et al. [41] and Singh et al. [47] reported that both treatment arms received non-surgical therapy with or without adjunctive antibiotics; Chen et al. [42] reported that both treatment arms received scaling and root planning only; while in the Telgi et al. [8] study only one arm received scaling. Of the two arm studies, two studies’ treatment included scaling and root planning only [44], [46], Koromantzos et al. [45] treatment included scaling and root planning plus extraction of hopeless teeth, and Sun et al. [40] treatment also included flap surgery (“when indicated”) and systemic Tinidazole plus ampicillin. The remaining studies included adjunctive treatment of chlorhexidine oral rinse [28] or topical use of minocycline ointment [43]. And five trials [28], [41]–[43], [45] reported the additional supra- or subgingival debridement at the post-treatment appointment.

## Discussion

There have been several excellent systematic reviews [35], [36] reported about the effect of periodontal treatment on glycemic control of diabetic patients, the previous studies used for analysis in the reviews except those couldn’t meet the inclusion criteria were included, and three new studies have been added. In all, ten randomized clinical trials were included in this review, with more than 3 months of follow-up period. The total number of subjects within the studies included in this review was 1135. And we could conclude from the current systematic review that non-surgical periodontal treatment for diabetic patients is beneficial in glycemic control and can reduce HbA1c levels by 0.36% 3 months after treatment, the effect at 6-month post-treatment is not obvious. It might present that non-surgical periodontal treatment may play a role on glycemic control for diabetic patient for just a period of time. But because of the difference of the treatment modalities the number of included studies is limited, the sample size is insufficient, and there is significant heterogeneity across studies, it is worthy of attention in future studies.

Although not all literature reported the related change of periodontal parameter, the available data extracted from the included studies was analyzed. The results showed that there was an overall 0.42 mm reduction in PPD and 0.34 mm reduction in CAL at 3-month post-treatment. It is generally considered that inflammation cytokines like C-reactive protein (CRP) may play an important role between diabetes and periodontitis [50], and a meta-analysis made by Teeuw et al. [51] find that periodontal treatment can significantly reduce the level of CRP. Katagiri et al. [43] revealed that there is a relationship between the change of CRP and HbA1c level. Therefore, we believe that periodontal therapy can certainly reduce systemic inflammation by improvement of the periodontal status, then impact the glycaemic control for a period of time. And when the inflammation is under control, the effect of periodontal therapy on glycaemic control may not obvious.

It is reported that although dental prophylaxis including removal of supragingival calculus and plaque can improve periodontal health, it cannot influence HbA1c levels [52]. So we considered the group in Boter et al. [41] received removal of supragingival calculus and plaque as the control group.

High risk of bias of the included studies especially the “blinding of participants and personnel”, should be explained by the fact that the intervention of the studies was periodontal treatment, such as scaling and root planning etc., while the control group without treatment, so it was difficult to mask the therapists and subjects.

Compared with the previous reports, there are some certain improvements in this study. Firstly, a phase 3, multi-center, randomized trial with a number of 514 participants was included [28]. Although the results of that trial was that non-surgical periodontal therapy did not improve glycaemic control in patients with type 2 diabetes, and was not exactly the same with this review, the difference made us to look for the reasons. Three significant reasons should be considered. One reason was that the characteristics of the participants in Engebretson et al. [28] and the previous studies were different. Although the participants were all diabetics, they were different in many aspects, such as ethnic and geographic, severity of periodontitis, compliance of the participants, oral hygiene habits, etc. Especially, the concern about the treatment of diabetes was different. Only the participants enrolled in Engebretson et al. [28] were reported under the supervision of the physician for the diabetes, and the change of diabetic medicine was monitored. Six of the other studies instructed the participants not to modify the medication or diet [40], [42]–[44], [46], [47], two did not reported the change of diabetic medication [8], [41] and the other one [45] reported that only 13.3% for treatment group and 10% for control group participants increased their insulin dosages. It was considered that many factors, including whether the diabetic patients were under the supervision of physicians, and the different regions, the level of medical institutions (medical centers or primary clinics), and the different types that the supervisors belong to [53], can influent the level of HbA1c. So we think that the concern about the treatment of diabetes may have an impact on the outcome of the studies. Although the author disproved, Chapple et al. [54] pointed out that almost 60% patients having HbA1c level less than 8% at the baseline in Engebretson et al. Another reason was that the sample size of Engebretson et al. [28] was much more bigger than the other included studies, and the range of standard deviation of the change from baseline of HbA1c was wide. This point also increases the difficulty for us to explain the heterogeneity. When the heterogeneity cannot be readily explained, random-effects model is one of the strategies for addressing heterogeneity according to the Cochrane handbook for systematic reviews of interventions (Version 5.1.0) [37]. So a random-effects model was used for the analysis. The other reason was that the treatment of the periodontitis was not completely the same with the other studies, this study did not use the adjunctive topical or systemic antibiotics [55], [56]. Since the effect of scaling and root planning with the adjunctive topical or systemic antibiotics on reduction of HbA1c level has been shown in several studies [41], [43], [47], we believe that it may be a potential factor affecting the results of the study.

Secondly, the follow-up period of the studies included in this review was more than 3 months, and all of the studies reported about the HbA1c values at the 3-month after treatment. Two studies [48], [49] included in the previous review reported on a 4-month follow-up outcome, and they were excluded.

Thirdly, One of the strengths of the present meta-analysis is that it takes into account the change of periodontal parameters at 3-month after treatment. It allows us to be more intuitive understanding of the effect of periodontal treatment on glycaemic control while improving periodontal status. The change of HbA1c for 6 months was also analyzed. The result can help us to understand the long-term effect of periodontal treatment on HbA1c level in subjects with diabetes and periodontitis.

There are also several limitations to this review. The first is that the effect of periodontal treatment on glycaemic control of different type of diabetes with periodontitis cannot be analyzed in subgroups. One study [41] included participants with type 1 diabetes. But the sample size was small, and the outcomes were not reported separately, so it was analyzed as a part of treatment and control group. Another limitation is that adjunctive treatments such as: topical or systemic antibiotics and topical antiseptics were taken in many trials. And the systemic antibiotics could potentially mask the effect of scaling and root planning on HbA1c [28]. And it was not reported in this study. The other limitation of the present meta-analysis is that several studies did not reported the standard deviation of the change of HbA1c, and the data included in the analysis were obtained by calculation. The last but not the least is that only one recent trials was included in this update systemic review.

In conclusion, we demonstrate that periodontal treatment leads a reduction of HbA1c in diabetic patients with periodontitis while the improvement of periodontal status for 3 months after treatment. But the treatment may have no obvious effect on glycemic control for diabetic patient for 6 months after treatment. So physicians and dentists should carefully interpret these results when they apply them in clinical practice. And we look forward to more large randomized controlled trials with clear intervention design.

## Supporting Information

Figure S1 **Forest plot presenting change in HbA1c (%) at 3-month (fixed-effects model).**
(EPS)

Figure S2 **Forest plot presenting change in PPD (mm) at 3-month (fixed-effects model).**
(EPS)

Figure S3 **Forest plot presenting change in CAL (mm) at 3-month (fixed-effects model).**
(EPS)

Figure S4 **Forest plot presenting change in HbA1c (%) at 6-month (fixed-effects model).**
(EPS)

Checklist S1 **Prisma Checklist.**
(DOC)

File S1 (DOCX)

Flow Diagram S1 (DOC)
