# Supplementary material for: The Effect of Periodontal Treatment on Hemoglobin A1c Levels of Diabetic Patients: A Systematic Review and Meta-Analysis
Source: PLoS One. 2014 Sep 25;9(9):e108412. doi: 10.1371/journal.pone.0108412 (PMC4177914; doi:10.1371/journal.pone.0108412)
Supplement: File S1 — (DOCX) [file pone.0108412.s006.docx]

**Results analyzed by fixed-effects model:**

**Change of HbA1c at 3-month**

Data on change of HbA1cwere available for analysis in 1135 patients enrolled in ten trials investigating the effect of non-surgical periodontal treatment on glycemic control of diabetic patients. The change of HbA1c for 3 months ranged from -0.86% to0.13%, with a mean change of HbA1c from baseline of -0.35% (95%CI: -0.40%, -0.31%), when it was analyzed by fixed-effects model.(Fig. S1)

**Change of periodontal parameters at 3-month**

Non-surgical periodontal treatment demonstrated a significant benefit on the periodontal status. Nine trials [8,28,40-44,46,47] reported the mean change of PPD in 1077 patients. The mean change of PPD from baseline to the 3-month after treatment was -0.42mm (95%CI: -0.48, -0.36)when it was analyzed by fixed-effects model.(Fig. S2); seven trials[28,40-42,44,46,47] reported the mean change of CAL in 968 patients, and the mean change of CAL 3 months after treatment was -0.41mm (95%CI: -0.50, -0.33) when it was analyzed by fixed-effects model.(Fig. S3)

**Change of HbA1c at 6-month**

Data on the change of HbA1c for 6 months in diabetic patients with periodontitis were available for 754 patients enrolled in four trials[28,41,42,45]. The change of HbA1c for 6 months ranged from -0.72% to 0.15%, and the result presented no statistical difference between the treatment and control groups (-0.19%, 95%CI: -0.37 to 0.00, *P*=0.05) when it was analyzed by fixed-effects model, and there might be heterogeneity in the change of HbA1cfor 6 months across studies (*P*=0.02, *I^2^*=68%). (Fig. S4)
