# Supplementary figures and images for: The Effect of Periodontal Treatment on Hemoglobin A1c Levels of Diabetic Patients: A Systematic Review and Meta-Analysis
Source: PLoS One. 2014 Sep 25;9(9):e108412. doi: 10.1371/journal.pone.0108412 (PMC4177914; doi:10.1371/journal.pone.0108412)

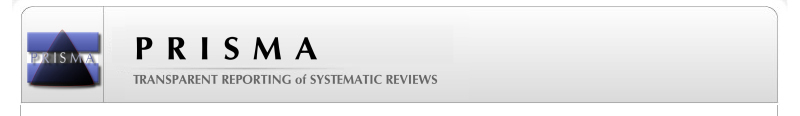
**PRISMA 2009 Flow Diagram**

**Screening**

**Included**

**Eligibility**

**Identification**

Supplement: Flow Diagram S1 — (DOC) [file pone.0108412.s007.doc]
